# Menin: Expanding and dichotomous roles in cancer

**DOI:** 10.18632/oncoscience.485

**Published:** 2019-08-23

**Authors:** Bryson W. Katona, Rebecca A. Glynn, Taylor A. Hojnacki, Xianxin Hua

**Affiliations:** ^1^ Division of Gastroenterology, University of Pennsylvania Perelman School of Medicine, Philadelphia, Pennsylvania; ^2^ Department of Cancer Biology, Abramson Family Cancer Research Institute, University of Pennsylvania Perelman School of Medicine, Philadelphia, Pennsylvania

**Keywords:** menin, colorectal cancer, SKP2, EGFR inhibitor

Menin, the protein product of the *MEN1* gene, is a ubiquitously expressed protein that lacks homology with other protein families, yet is highly conserved among various species [1]. Menin primarily resides in the nucleus, where it serves as a scaffold for epigenetic regulators [1, 2]. While much is known about menin and its diverse roles in numerous cellular processes, there remains much to be discovered, especially with regard to its role in cancer.

## Menin and cancer

The first association of menin with cancer was recognized through the study of multiple endocrine neoplasia type 1 (MEN1) syndrome [1]. Individuals with the autosomal dominant MEN1 syndrome have a germline mutation in the *MEN1* gene that leads to increased risk of pituitary, parathyroid, and pancreatic neuroendocrine tumors. For these particular tumors menin clearly serves as a tumor suppressor, however this tumor suppressor function is not global amongst all types of cancers. In fact, menin serves as a contextual tumor promoter in MLL-fusion leukemia. Menin promotes MLL- and MLL-fusion protein-induced increases in H3K4-trimethylation and HOX gene expression, which is critical for leukemogenesis [3]. Consequently, inhibitors of menin (MIs) are effective in the treatment of this subset of leukemias in preclinical models [3]. Additionally, menin serves as a contextual tumor promoter in prostate cancer, where menin/MLL acts as an important co-activator of the androgen receptor [4]. Consistently, MIs also demonstrate anti-neoplastic effects in prostate cancer [4].

**Figure 1:**
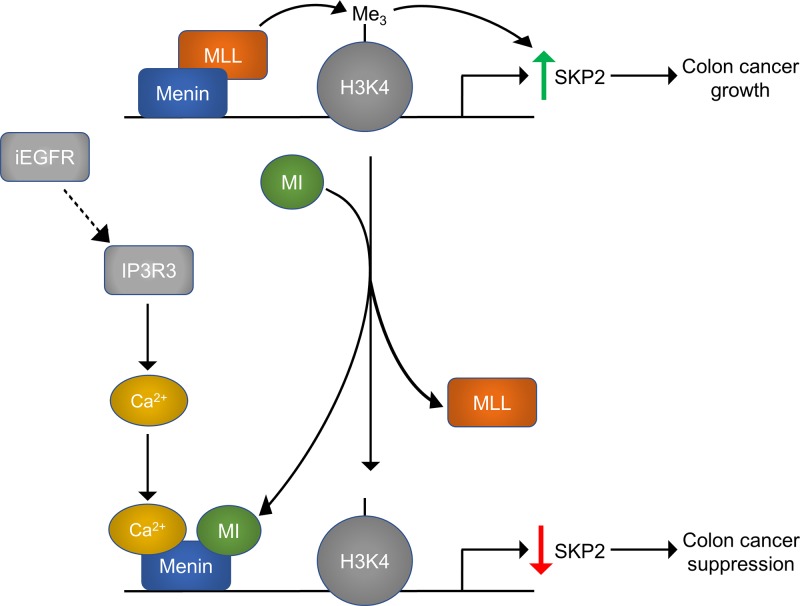
The combination of a menin inhibitor (MI) and small molecule EGFR inhibitor (iEGFR) synergistically suppress CRC through repression of SKP2 transcription

Our group recently showed that menin is over-expressed in colorectal cancer (CRC) compared to normal colonic mucosa [5]. CRC cell growth does not depend on functional menin, unlike MLL-fusion leukemias and prostate cancer. However, we found menin is important for CRC resistance to small molecule EGFR inhibitors (iEGFR) such that combined treatment of CRC with a MI and iEGFR led to synergistic cell death through transcriptional suppression of the E3 ubiquitin ligase SKP2 (Figure 1). There are several important points to highlight from these recent findings. First, this synergy is independent of EGFR, and is instead dependent on iEGFR-induced increases in cytosolic calcium. We found that iEGFRs induce activation of inositol trisphosphate receptor 3 (IP3R3), causing release of ER calcium to the cytoplasm. Second, menin is critical for maintaining expression of pro-tumorigenic SKP2, which is necessary for ubiquitin-mediated degradation of anti-proliferative proteins, such as p27. Menin binds the *SKP2* promoter and facilitates MLL-mediated H3K4-trimethylation to maintain SKP2 expression. Third, menin directly binds calcium, and increased cytosolic calcium reduces menin's ability to promote transcription of SKP2. Taken together, these results reveal a novel role for menin in regulating CRC and a new mechanism to potentially develop novel therapeutic combinations for CRC.

## Challenges and future directions

While menin is increasingly recognized as being important in regulating cancer, whether menin suppresses or promotes growth appears to be highly tissue specific. The role of menin in other cancers apart from CRC, prostate, MLL-fusion leukemias, and MEN1 syndrome spectrum tumors is largely unknown and certainly merits further investigation. Furthermore, there is a clear need to better understand the in-depth biology of menin. In our recent work, we reveal a novel cross-talk between menin and EGFR inhibitor-induced calcium release that converges on regulation of SKP2 transcription [5]. There are undoubtedly numerous other menin-related signaling pathways that remain to be discovered, and these pathways will likely be integral in maintaining the epigenomes of various types of cancers, as well as the epigenomes in various cognate normal tissues. Additionally, how calcium may alter the function of menin and menin-mediated epigenetic regulation should be further investigated.

Given the increasing number of cancers where menin serves pro-tumor roles, developing effective menin inhibitors is of the utmost importance. Small molecule MIs have been developed by several groups [3, 6], and some of these MIs are orally bioavailable and display efficacy in mouse models [3]. Recent work on a menin/MLL inhibitor KO-539 by Kura Oncology has led to the first FDA approval of an investigational new drug (IND) application for a small molecule MI [7]. This approval will likely lead to an initial phase I study in humans, which is an important first step in working to translate MIs from the bench to the bedside.

Our work linking menin to CRC highlights the importance of investigating the use of MIs with other anti-neoplastic agents. iEGFRs alone have not demonstrated clinical success in treating CRC [8, 9], however, if a MI can be developed to the clinical market, it is important to readdress whether patients with metastatic CRC may benefit from combination therapy with an iEGFR and MI. Furthermore, there may be benefit with combining MIs with iEGFRs in other cancers where iEGFRs are currently clinically approved and utilized in treatment, such as in non-small cell lung cancer with activating EGFR mutations [10]. Additionally, as MIs synergized with the EGFR-independent effects of iEGFRs [5], it is important to remember that many approved drugs have “off-target” effects, and that exploiting these “off-target” effects can potentially be a successful strategy to increase their efficacy.

## Conclusion

While recent exciting work focused on better understanding the role of menin in cancer, much remains to be discovered. Important priorities necessary to advance the field forward include understanding the unique role of menin in other cancers, elucidating the complex cellular and biochemical functions of menin, and developing effective menin inhibitors that can be translated to clinical trials.
